# Antioxidant activity of herbaceous plant extracts protect against hydrogen peroxide-induced DNA damage in human lymphocytes

**DOI:** 10.1186/1756-0500-6-490

**Published:** 2013-11-26

**Authors:** Kuan-Hung Lin, Yan-Yin Yang, Chi-Ming Yang, Meng-Yuan Huang, Hsiao-Feng Lo, Kuang-Chuan Liu, Hwei-Shen Lin, Pi-Yu Chao

**Affiliations:** 1 Graduate Institute of Biotechnology, Chinese Culture University, Taipei 11114, Taiwan; 2 Graduate Institute of Applied Science of Living, Chinese Culture University, Taipei 11114, Taiwan; 3 Research Center for Biodiversity, Academia Sinica, Nankang, Taipei 11106, Taiwan; 4 Department of Horticulture and Landscape Architecture, National Taiwan University, Taipei 11111, Taiwan; 5 Taoyuan District Agricultural Research and Extension Station, Taoyuan 327 Taiwan; 6 Department of Nutrition and Health Sciences, Chinese Culture University, Taipei 11114, Taiwan

**Keywords:** Herbaceous plants, Antioxidant activity, Flavonol, Comet assay, Human lymphocytes

## Abstract

**Background:**

Herbaceous plants containing antioxidants can protect against DNA damage. The purpose of this study was to evaluate the antioxidant substances, antioxidant activity, and protection of DNA from oxidative damage in human lymphocytes induced by hydrogen peroxide (H_2_O_2_). Our methods used acidic methanol and water extractions from six herbaceous plants, including *Bidens alba* (BA), *Lycium chinense* (LC), *Mentha arvensis* (MA), *Plantago asiatica* (PA), *Houttuynia cordata* (HC), and *Centella asiatica* (CA).

**Methods:**

Antioxidant compounds such as flavonol and polyphenol were analyzed. Antioxidant activity was determined by the inhibition percentage of conjugated diene formation in a linoleic acid emulsion system and by trolox-equivalent antioxidant capacity (TEAC) assay. Their antioxidative capacities for protecting human lymphocyte DNA from H_2_O_2_-induced strand breaks was evaluated by comet assay.

**Results:**

The studied plants were found to be rich in flavonols, especially myricetin in BA, morin in MA, quercetin in HC, and kaemperol in CA. In addition, polyphenol abounded in BA and CA. The best conjugated diene formation inhibition percentage was found in the acidic methanolic extract of PA. Regarding TEAC, the best antioxidant activity was generated from the acidic methanolic extract of HC. Water and acidic methanolic extracts of MA and HC both had better inhibition percentages of tail DNA% and tail moment as compared to the rest of the tested extracts, and significantly suppressed oxidative damage to lymphocyte DNA.

**Conclusion:**

Quercetin and morin are important for preventing peroxidation and oxidative damage to DNA, and the leaves of MA and HC extracts may have excellent potential as functional ingredients representing potential sources of natural antioxidants.

## Background

Herbaceous plants have a long history of use as medicine, food, and a variety of daily needs. Many epidemiological studies suggest that an increased consumption of several medicinal plants containing antioxidants can protect against DNA damage and carcinogenesis, and often exhibit a wide range of pharmacological activities such as antiflammatory, anti-bacterial, and anti-fungal properties [1]. Flavonoids have strong antioxidant efficiencies and are common in leafy vegetables. Trolox, for example, is a water-soluble derivative of vitamin E that blocks DNA fragmentation in irradiated MOLT-4 cells, a human lymphocytic leukemia line [2]. Hence, a number of phytochemicals commonly used in research have antioxidant activity that can protect cells from reactive oxygen species (ROS)-mediated DNA damage that results in mutation and subsequent carcinogenesis [3,4]. Cao *et al.*[5] indicated that increased consumption of vegetables and fruits increases the plasma antioxidant capacity in humans. Some common vegetables like purple-leaved sweet potato and the outer layers of purple onions abound in quercetin and myricetin, which scavenge 2, 2-diphenyl-1-picrylhydrazyl (DPPH), superoxide, and hydroxyl radicals, and inhibit lipid peroxidation [6]. The search for phytochemicals and dietary compounds with potent antioxidant and otherwise preventive properties continues to be of great importance in the search for remedies against free radical-mediated diseases. There is great interest in the use of potent dietary antioxidants in preventive strategies for applications ranging from the prevention of oxidative reactions in foods and pharmaceuticals to the role of ROS in chronic degenerative diseases [7].

In recent years, increasing attention has been paid by consumers to the health and nutritional benefits of herbaceous plants. Some herbs, such as pilosa beggarticks (*Bidens alba* L. var. *minor*) (BA), Chinese wolfberry (*Lycium chinense* Mill.) (LC), wild mint or corn mint (*Mentha arvensis* L. var. piperascens Malinv.) (MA), Asiatic plantain (*Plantago asiatica* L.) (PA), heartleaf (*Houttuynia cordata* Thunb.) (HC), and Asiatic centella (*Centella asiatica* L. Urban) (CA) are favored as functional herbals. Some of the health effects of herbaceous plants have been reported to include antioxidation [8-10], anti-inflammation [11], and blood pressure reduction [12]. In animal experiments, Chinese wolfberry, heartleaf, Asiatic plantain, Asiatic centella, and pilosa beggarticks showed special detoxification and anti-inflammatory effects [8,9,11,13,14]. Particularly, HC, LC, and CA showed antioxidant activities [8,9]. Asiatic centella increased the activity of antioxidant enzymes such as superoxide dismutase, catalase, and glutathione peroxidase, and enhanced the concentration of vitamin C and vitamin E in new tissues during wound healings [13]. Both HC and BA were reported to have anti-inflammatory functions due to their quercetin and luteolin content [8,11]. Furthermore, LC and BA can reduce the injury to liver cells from CCl_4_[9,13]. Pilosa beggarticks also functions as an anti-fungal and anti-bacterial agent, and lowers high blood pressure [12]. Several herbs are consumed to protect against common, serious diseases such as cardiovascular and cerebrovascular events, cancer, and other age-related degenerative diseases [15]. These protective effects are considered, in large part, to be related to the various antioxidants contained in them. Evidence that free radicals cause oxidative damage to lipids, proteins, and nucleic acids is overwhelming. Antioxidants, which can inhibit or delay the oxidation of an oxidizer in a chain reaction, would therefore seem to be important in preventing these diseases [16]. Prevention from oxidative stress might be achieved by the uptake of antioxidants. Polyphenols and flavonols can act as antioxidants in two ways: by scavenging free radicals and chelating redox active metal ions (direct antioxidant activity), and by inducing cellular antioxidant defense and repair. These benefits have significantly contributed to their antioxidant activity and have stimulated research into the content, ability, capacity, and function of antioxidant systems in herbaceous plants. Polyphenolic and flavonol substances are the most common compounds in herbs having strong antioxidant activity [6]. Previously, we also demonstrated that purple-leaved sweet potato exhibits free radical scavenging and has high polyphenolic content [17]. Although a variety of medicinal herbs are known to be potent sources of polyphenolic and flavonol compounds, studies that isolate polyphenols, evaluate their antioxidative effects, and determine their efficacy or ability to prevent oxidative damage to DNA are either scarce or little known. The bioactive components of these herbal plants might be responsible for anti-cancer effects through growth inhibition and apoptosis in human chronic myeloid leukemia K562 cells [18]. The objective of this study was to isolate, identify, and evaluate the antioxidant components, antioxidant activity, and extent to which methanolic acid hydrolysates and water extracts of six herbaceous plants could protect DNA in human lymphocytes from oxidative damage induced by H_2_O_2_. Our study explores the relationships between the composition and content of flavonols and polyphenol having antioxidant efficiency, and the prevention of DNA oxidative damage afforded by the herbaceous plants.

## Methods

### Chemicals and reagent

Methanol, ethanol, hydrochloric acid, di-sodium hydrogen phosphate, potassium dihydrogen phosphate, formic acid, sodium chloride (NaCl), potassium chloride (KCl), Tris–HCl, Tris (hydroxymethyl) aminomethane (Tris base), dimethyl sulfoxide (DMSO), ethylenediamine tetraacetic acid (EDTA), Trolox, and butylated hydroxyltoluene were purchased from Merck (Darmstadt, Germany). Linoleic acid, d-glucose, calcium chloride dihydrate, sodium lauryl sarcosinate, gallic acid, 2,2-azino-bis-(3-ethylbenzothiazoline-6-sulfonicacid) (ABTS), peroxidase, H_2_O_2_, sodium carbonate (Na_2_CO_3_), tetrazolium/formazan, Folin-Ciocalteau reagent, and ethidium bromide were procured from Sigma Chemical (St Louis, MO, USA). Myricetin, morin, quercetin, kaempferol, cynidin, and malvidin were obtained from ROTH (Rheinzabern, Denmark). Ficoll-Paque was acquired from Amersham Biosciences (Uppsala, Sweden). Low-melting gel agrose and Triton X-100 were purchased from BDH (Poole, England). Normal-melting gel agarose was purchased from Pantech Instruments (Darmstadt, Germany). AIM V serum-free lymphocyte medium was purchased from Gibco Invitrogen (Carlsbad, CA, USA).

### Herbaceous plants

The tested plants were *Bidens alba* L. var. *minor*, *Lycium chinense* Mill., *Mentha arvensis* L. var. piperascens Malinv., *Plantago asiatica* L., *Houttuyni acordata* Thunb., and *Centella asiatica* L. Urban. These were generously provided by Dr. Kuang-Chuan Liu, Taoyuan District Agricultural Research and Extension Station Council of Agriculture, Executive Yuan, Taiwan.

### Preparation of plant extracts

The plants were weighed, lyophilized, and ground to powder. Each lyophilized powder was extracted by distilled deionized (dd)H_2_O. The extraction mixture was then heated to 90°C in a steam bath and refluxed for 2 h, allowed to cool in a refrigerator, sonicated for 5 min, and diluted to 50 mL with ddH_2_O to prepare the final extract. These water extracts were ready for the comet assay. For high-performance liquid chromatography (HPLC), only the edible portions of plants were weighed, lyophilized, and ground into powder. Lyophilized vegetable powders were prepared according to Justesen *et al*. [19] with modifications as follows: 10 ml of 62.5% aqueous methanol containing butylated hydroxyltoluene (2 g/L) were added to 1.25 g of lyophilized samples, followed by adding 5 mL of 6 M HCl to bring total volume up to 12.5 mL. The final mixture consisted of 1.2 M HCl in 50% aqueous methanol. The extraction mixture was thereafter heated to 90°C in a steam bath and refluxed for 2 h, allowed to cool in a refrigerator, sonicated for 5 min, and diluted to 50 mL with methanol to form the final extract. The acid hydrolysates methanolic extract was ready for high-performance liquid chromatography (HPLC), inhibition of conjugated diene formation in the linoleic acid assay, TEAC assay, and comet assay.

### Polyphenol assay

Polyphenol content was determined according to the method of Taga *et al*. [20]. Briefly, standard gallic acid and an aliquot of methanolic extract were diluted with an ethanol/water (60:40, v/v) solution containing 0.3% HCl. Two mL of 2% Na_2_CO_3_ was mixed into each sample of 100 μL and allowed to equilibrate for 2 min before adding 50% Folin-Ciocalteau reagent. Absorbance at 750 nm was measured at room temperature. The standard curve of gallic acid was used to calculate polyphenol levels.

### Flavonols analysis by HPLC

One mL of acid hydrolysates methanolic extract was filtered through a 0.45 μm filter prior to 20 μL being injected into the HPLC. Samples were analyzed with a SpectraSYSTEMUV6000LP Photodiode Array Detection System (Thermo Separation Products, San Jose, USA) and an ODS column (250 × 4.6 mm, 5 μm; YMC, Kyoto, Japan). The mobile phase consisted of methanol–water (30:70, v/v) with 1% formic acid and 100% methanol. The gradient was 25 - 74% methanol in 40 min at a flow rate of 0.75 mL/min. Spectra were recorded at 365 nm for flavonols [19].

### Inhibition of conjugated diene formation in linoleic acid emulsion autoxidation system

The inhibition of conjugated diene formation was determined according to Mitsuda *et al*. [21]. Briefly, an aliquot of 0.1 mL of diluted plant acidic methanolic extract or blank was added to 2 mL of 10 mM linoleic acidemulsion (pH 6.6), mixed well, and incubated at 37°C for 15 h. A sample of 0.2 mL for 0 and 15 h incubation periods were mixed with 7 mL of 80% methanol, followed by measuring the absorbance at 234 nm.

### Trolox equivalent antioxidant capacity (TEAC) analysis

The total antioxidant capacity of hydrophilic and lipophilic antioxidants was determined using the horseradish peroxidase catalyzed oxidation of 2,2-azino-bis-(3-ethylbenzothiazoline-6-sulfonicacid) (ABTS) [22]. The reaction mixture contained 0.5 mL of 1000 μM ABTS (in ddH_2_O) and 3.5 mL of 100 μM H_2_O_2_ (in ddH_2_O). The reaction was started by adding 0.5 mL of 44 U/mL peroxidase (in 0.1 M PBS). After 1 h, 0.05 mL of plant acidic methanolic extracts were added to the mixture. After 5 min, absorbance was measured at 730 nm. Trolox (TR) was used as a standard, and the total antioxidant capacity of plant extracts were measured as mM TR equivalent.

### Isolated human peripheral blood lymphocytes

Fasting blood samples were obtained from six donors, including four male and two female healthy non-smokers, 24–48 years old. Fresh venous blood (20–30 mL) was collected in lithium heparin tubes (Becton- Dickinson) from volunteers, and lymphocytes were isolated using a separation solution kit supplemented with Ficoll-Paque Plus lymphocyte isolation sterile solution (Pharmacia Biotech, Sweden) [23]. Cells were harvested within 1 day of taking the blood samples and cultured with AIM V serum-free lymphocyte medium (Gibco Invitrogen, USA) in a humidified atmosphere of 5% CO_2_ in air at 37°C for 24 h.

### Cell viability testing

After culturing, lymphocytes were exposed to each of six different plant acidic methanolic and water extracts. Each lymphocyte was treated with three concentrations of plant acidic methanolic and water extracts (25, 50, and 100 μg/mL) for 30 min at 37°C. DNA damage was induced by exposing lymphocytes to H_2_O_2_ (10 μM) for 5 min on ice to minimize the possibility of cellular DNA repair after H_2_O_2_ injury. Cells were centrifuged (100 *g* for 10 min), washed, and re-suspended in the same medium as the comet assay. All experiments were carried out in triplicate. Cell viability was tested using the tetrazolium/formazan (MTT) assay [24] both prior to and after treatment with plant extracts or H_2_O_2_.

### DNA single strand break damage estimation using the comet assay

The standard comet assay was performed as described in Szeto *et al*. [3], with acidic methanolic and water extracts from these six herbal plants being used for this study. Cultured lymphocytes (10^5^ cells/mL) were embedded in 75 μL of 1% low-melting-point agarose on a microscope slide (precoated with agarose) at 37°C. The gel was allowed to set at 4°C, and cells were lysed for a period of at least 2 h in lysis buffer at 4°C. Cells were then alkaline-unwound, following which electrophoresis was carried out using the electrophoresis buffer at 4°C for 15 min at 25 V with the current adjusted to 300 mA. All steps were conducted under dim light to prevent the occurrence of additional DNA damage. Following electrophoresis, slides were neutralized with neutralization buffer and stained with ethidium bromide. The comet-like images resulting from the extension of DNA were scored as a reflection of the single strand breaks under a fluorescence microscope (Zeiss-Axiovert 100, Zeiss, Germany). Triplicate slides were prepared for each experimental point sample, and 50 comet-like images selected at random per slide were evaluated to determine average DNA damage values. A computerized image analysis system (VisCOMET 1.6, Impuls GmbH, Germany) was employed to determine various comet parameters, and used to analyze DNA damage by tail DNA% [(total brightness of tail area / total brightness of total area) × 100%] and tail moment (tail length × tail DNA%). Inhibition percentage of tail DNA% and tail moment were calculated relative to the 10 μM H_2_O_2_ treated group.

### Statistical analysis

Data were analyzed by one-way analysis of variance (ANOVA), and the significance between means by the least significant difference (LSD) test. Pearson’s linear correlation was also determined. Means of three replicates were reported.

## Results

### Antioxidant composition and antioxidant activity

Table 1 documents the content of polyphenol in the leaves of tested plants. Polyphenols were significantly abundant in both BA (32.90 mg gallic acid/g DW) and CA (32.03 mg gallic acid/g) compared to other plants. Table 2 presents varied amounts of flavonols ranging from 53.33 to 3200 μg/g DW in the acidic methanolic extract of the studied plants. BA and CA were also rich in myricetin, at levels of 1133.33 and 960.00 μg/g DW, respectively. Morin was present only in MA, CA, and BA plants at a level of 2000.00, 600.00, and 573.33 μg/g DW. Quercetin was abundant in HC (3200.00 μg/g DW), while CA followed at a level of 533.33 μg/g DW. Kaempferol was abundant in CA at a level of 853.33 μg/g DW, but LC and PA did not contain any kaempferol at all. Thus, these species displayed variations in their polyphenol and flavonol levels.

**Table 1:** The content of polyphenol in tested herbaceous plants

**Sample**	**Ployphenol (mg gallic acid/g DW)**
BA	32.90^a^
LC	25.31^b^
MA	21.24^c^
PA	24.31^b^
HC	19.82^d^
CA	32.03^a^

Means with different superscripts (^a~d^) are significantly different, *p* < 0.05.

DW: dry weight.

**Table 2:** The content of flavonols in acidic methanolic extracts of tested herbaceous plants

**Flavonols (μg/g DW)**				
**Sample**	**Myricetin**	**Morin**	**Quercetin**	**Kaempferol**
BA	1133.33^a^	573.33^c^	93.33^d^	66.67^c^
LC	320.00^d^	N.D.	N.D.	N.D.
MA	480.00^c^	2000.00^a^	53.33^e^	333.33^b^
PA	253.33^e^	N.D.	213.33^c^	N.D.
HC	146.67^f^	N.D.	3200.00^a^	53.33^c^
CA	960.00^b^	600.00^b^	533.33^b^	853.33^a^

Means within a column with different superscripts (^a~f^) are significantly different, *p* < 0.05. N.D.: not detectable.

The inhibition of linoleic acid peroxidation was observed to be significantly higher in PA and BA at both 25 and 50 μg/mL of plant extracts (Table 3). Furthermore, significantly higher percentages of conjugated diene inhibition were detected in PA (79.31) and CA (77.61) compared to MA (70.31) at 100 μg/mL of the extract. Hence, each species showed significant differences in inhibition percentages of conjugated dienes at various extract concentrations.

**Table 3:** Inhibition percentage of conjugated diene formation in the linoleic acid emulsion autoxidation system treated with various concentrations of methanolic acid hydrolysates of herbaceous plants

**Inhibition percentage**			
**Sample**	**25 μg/mL**	**50 μg/mL**	**100 μg/mL**
BA	49.14^ab^	55.71^b^	73.72^b^
LC	40.14^c^	52.31^c^	74.69^b^
MA	46.23^b^	56.20^b^	70.31^c^
PA	54.50^a^	68.37^a^	79.31^a^
HC	45.49^b^	59.61^b^	72.99^b^
CA	41.11^c^	58.63^b^	77.61^a^

Means within a column with different superscripts (^a~c^) are significantly different, *p* < 0.05.

Plant extracts from the six species showed antioxidant activities, proving their capacity to scavenge the ABTS radical-cation. The antioxidant activity in methanolic acid hydrolysate extracts of leaf tissues of studied species were expressed in Trolox Equivalent Antioxidant Capacity (TEAC) (Table 4). HC showed a significantly higher TEAC value (231.16 mM) than other species.

**Table 4:** The TEAC values of acidic methanolic extracts in the investigated herbaceous plants

**Samples (100 μg/mL)**	**TEAC (mM Trolox)**
BA	184.61^b^
LC	117.44^e^
MA	146.44^c^
PA	142.49^c^
HC	231.16^a^
CA	132.80^d^

Means within a column with different superscripts (^a~e^) are significantly different, *p* < 0.05.

### Effects of acidic methanolic and water extracts from herbaceous plants on H_2_O_2_-induced DNA damage to lymphocytes

**Figure 1:**
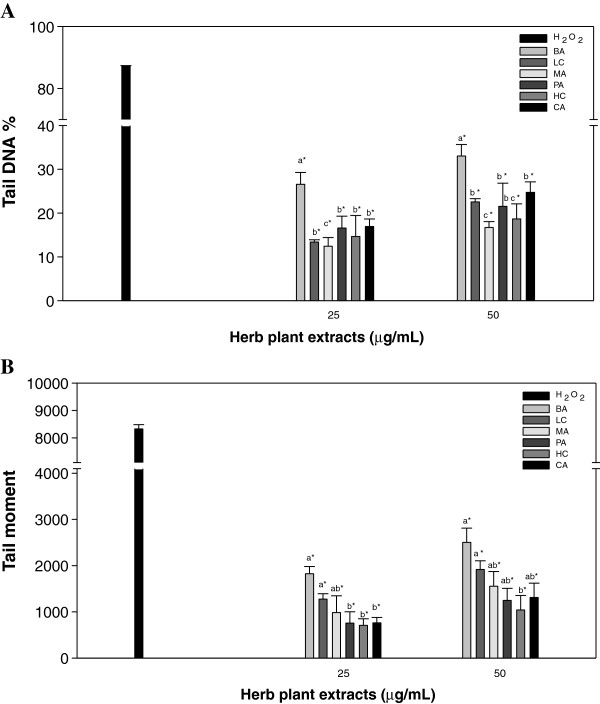
**Effects of various acidic methanolic extracts from six herbaceous plants on H**_**2**_**O**_**2**_**-induced DNA damage to lymphocytes.** Tail DNA% **(A)** and tail moment **(B)** were measured after exposure to tested compounds at 25 and 50 μg/mL of extract. , BA;, LC; , MA; , PA; , HC; ■, CA. Values with different letters differ significantly with regard to oxidative damage when comparing different plant extracts; *p < 0.05 refers to differences in oxidative damage as compared with 10 μM H_2_O_2_-alone (■) treatment.

Lymphocytes were exposed to each of three different herbal extracts at three concentrations (25, 50, and 100 μg/mL) for 30 min at 37°C. DNA damage was induced by exposing lymphocytes to H_2_O_2_ (10 μM) for 5 min on ice. At two lower levels, no extracts were cytotoxic at the concentrations used, with > 98% of cells remaining viable [25]. Therefore, concentrations only at 25 and 50 μg/mL were chosen for the comet assay. The comet assay was performed to determine the DNA damaging activity of the plants as it is a sensitive method for monitoring single strand DNA breaks at the single cell level. Any DNA damage is represented as tail DNA% and tail moment. The effects of pretreatment of the six tested extracts on 10 μM H_2_O_2_-induced DNA oxidative damage in human lymphocytes are presented in Figure 1. Tail DNA% demonstrated that MA had a significantly greater level of protection against H_2_O_2_ exposure than lymphocytes that were exposed to other tested compounds at two doses (25 and 50 μg/mL) (Figure 1A). The maximum protective effect of lymphocyte pretreatment was observed with pretreatment by 25 μg/mL MA, exhibiting 12.43% of tail DNA% compared to the rest of treated samples. Furthermore, at lower concentrations, all tested samples had lower tail DNA%, indicating better inhibition efficacies. The MA extract at the 50 μg/mL was significantly lower than the rest of treated samples, except for HC extract. Tested plants showed at least 707.53 and 1040.63 of tail moment in HC extract at 25 and 50 μg/mL levels compared to the rest of the acidic methanolic extract samples (Figure 1B).

**Figure 2:**
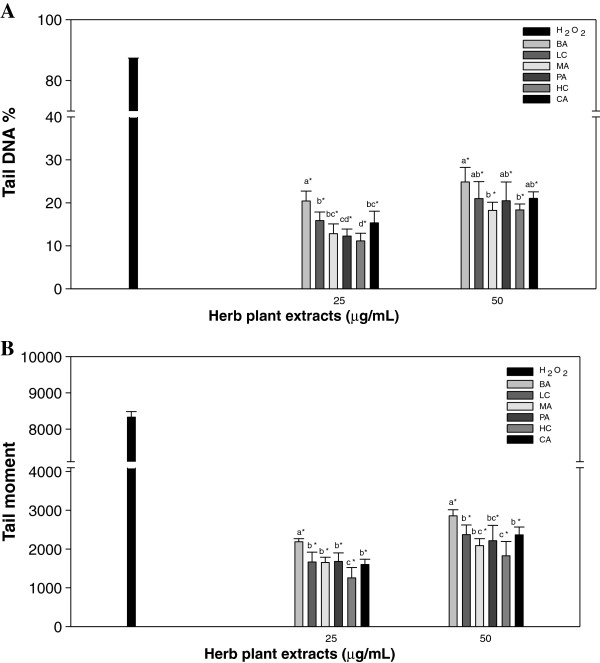
**Effects of various water extracts from six herbaceous plants on H**_**2**_**O**_**2**_**-induced DNA damage to lymphocytes.** Tail DNA% **(A)** and tail moment **(B)** were measured after exposure to tested compounds at 25 and 50 μg/mL of extract. , BA; , LC; , PA; , HC; ■, CA. Values with different letters differ significantly with regard to oxidative damage when comparing different plant extracts; *p < 0.05 refers to differences in oxidative damage as compared with 10 μM H_2_O_2_-alone (■) treatment.

HC had the lowest % tail DNA at 11.14% in 25 μg/mL of water extract (Figure 2A). Both HC (18.36%) and MA (18.25%) extracts at 50 μg/mL had lowest % tail DNA compared to the rest of the water extract of samples. Moreover, HC also had a significantly lower tail moment (1255.40 ~ 1826.10) than the rest of the water extracts at the same doses (Figure 2B). Hence, the DNA damage induced by H_2_O_2_ was significantly high as compared to the treated extracts, which had 87.26 in tail DNA% and 8328.84 in tail moment.

## Discussion

### Antioxidant composition and antioxidant activity

Plant leaves are rich in flavonols and other pigments. BA and CA plants contain higher polyphenol levels than the other plants tested (Table 1). Antioxidant activities are known to increase proportionally to the polyphenol content, mainly due to their redox properties [1]. Among the diverse roles of polyphenols, they protect cell constituents against destructive oxidative damage, thus limiting the risk of various degenerative diseases associated with oxidative stress and tending to be potent free radical scavengers. Their ability to act as antioxidants depends on their chemical structure and ability to donate/accept electrons, thus delocalizing the unpaired electron within the aromatic structure [26]. Phenolic compounds are known as radical scavengers or radical-chain breakers, and they strongly eliminate oxidative free radicals. Quercetin and morin are the principal flavonol constituents in HC and MA plants, respectively (Table 2). These antioxidant compounds may account for the high antioxidant power of the plants in the present study. Quercetin, kaempferol, morin, and myricetin are the most common flavonols, and are the most widely distributed flavonoids in plant leaves. Quercetin, the most abundant flavonoid in the human diet, is an excellent free radical scavenging antioxidant [27]. Polyphenol and flavonol contents found in the extracted plants (Tables 1, 2) were much lower than those in our previous study where purple-leaved sweet potato appeared to have higher contents [28]. A possible reason is the usage of different extraction methods. In fact, different results were obtained from the water and acidic methanolic extracts, and especially from the water extracts. The antioxidant composition and activities of herbal plants cannot be evaluated by a single method due to the complex nature of plants, in which pigments and phytochemicals have specific functions. Therefore, several methods should be employed to evaluate the total antioxidant effects of any plant. Antioxidant compounds presented in plant extracts are therefore multi-functional and their activities and mechanisms of action would largely depend on the composition and conditions of the test system.

Compared to the inhibition percentage of conjugated diene formation in the linoleic acid emulsion autoxidation system of tested samples, PA exhibited relatively higher effectiveness than the others at all extract concentrations (Table 3). The tested vegetables showed >70% inhibition of linoleic acid peroxidation in 100 μg/mL extracts, and PA in particular exhibited the highest inhibition of linoleic acid peroxidation, up to 79.31%. Therefore, all tested plants were effective inhibitors and exhibited better inhibition efficacy at higher concentrations. Previously, we demonstrated that water and methanolic extracts from PA both had higher antioxidant activity, and that the antioxidant activity of PA was equivalent to 10^-4^ M of Trolox in preventing conjugated diene formation during linolic acid peroxidation at 62.5 μg/mL of methanolic extract [29]. The polyphenol content of methanolic extracts was significantly correlated with the delay of the lag phase of low-density lipoprotein (LDL) treated with methanolic extracts. Moreover, the polyphenol content of the methanolic extract of herbaceous plants was significantly correlated with scavenging DPPH radical activity and ferric reducing power [29].

We measured the direct antioxidant activity of acidic methanolic extracts by TEAC assay, reflecting the major mechanisms of antioxidant action for evaluating their relevance to cell protection (Table 3). Jastrzebski *et al.*[30] reported that prolipid, a mixture of herbs used as a plasma lipid lowering medicine, had strong antioxidant activity. The correlation coefficients between the polyphenols, flavonoids, and TEAC of prolipid water extracts were 0.97 and 0.90, respectively. They concluded that the content of polyphenol in prolipid was the main contributors to the overall antioxidant activity of prolipids. The antioxidant activity of leaf extracts from CA was found to have a direct linear relationship between total phenolic content and total antioxidant activity, indicating that phenolic compounds might be the major contributors to the antioxidant activities of CA extracts [31]. Chung *et al.*[29] reported that PA, BA, CA, Curled Spearmint, MA, and Mesona had higher total phenolic contents compared to LC and Taiwan lily, and that CA and PA had higher antioxidant activity. In this study, we found that HC and CA contained abundant quercetin while MA and CA were rich in morin and kaempferol, respectively. Additionally, BA and CA had significantly higher levels of myricetin than other tested samples (Table 2). These different pigments may exhibit effective antioxidant activity alone or synergistically, and are a likely cause of cultivar differences. Wang *et al.*[32] demonstrated that the H donation potential was quercetin > myricetin > morin > kaempferol, indicating that the presence of a 3′,4′-catechol moiety in the B ring correlated with high activity. Moreover, the structural peculiarity of di-OH in the B ring obviously rendered quercetin and morin more potent as ROS inhibitors than myricetin and kaempferol, which have tri- and mono-OH in the B ring, respectively. The unclear relationship between antioxidant activity and flavonol extracts indicates that the structure prerequisite to reinforce free radical scavenging activity may vary with the type of free radical. The synergisms among antioxidants make antioxidant activity dependent not only on the concentration, but also might be due to their structures and interactions among antioxidants [33]. The accumulation of flavonoid metabolites in the appropriate target site is probably required to exert their antioxidant activity. The polyphenol-rich plant extracts exhibited distinct cell-free antioxidant activity (TEAC) according to their levels of polyphenol and flavonols, with distinct antioxidant activity strongly accounting for the antioxidant activity of the extracts. HC plants containing 3200 μg/g DW quercetin (Table 2) exhibited the highest TEAC value (231.16 mM) within the tested extracts (Table 4).

### Estimation of DNA single strand break damage from exposure to acidic methanolic and water extracts

Quercetin was found to protect against H_2_O_2_-induced DNA damage in human lymphocytes at 10 μM [34] and at 3.1 to 25 μM [35]. However, it was found to induce DNA damage in human lymphocytes at higher concentrations, such as 100 μM or above [34]. Similarly, myricetin was also found to decrease oxidant-induced DNA damage at 100 μM, although α–tocopherol and β–carotene did not behave similarly. This might be due to the dihydroxy structure of quercetin and myricetin being essential for protecting DNA against hydrogen peroxide [34]. No such hydroxyl groups are present in the tocopherol molecule. This may reflect structure/activity relationships or the localization of the antioxidant relative to free radical generation within cells. Noroozi *et al*. [36] demonstrated that, in addition to quercetin, kaempferol could also inhibit H_2_O_2_-induced DNA strand breaks in human lymphocytes. Zhu and Loft [37] reported that aqueous extracts of cooked and autolysed Brussels sprouts decreased DNA strand breaks in human lymphocytes, with the maximum inhibition being 38 and 39% at cooked and autolysed extract levels of 10 μg/mL and 5 μg/mL, respectively, with the inhibition effect decreasing at increasing concentrations up to 100 μg/mL. Quercetin-rich onions showed increased resistance of lymphocytic DNA to *ex vivo-*induced oxidation [15]. In addition, several types of natural antioxidants, including flavonols and polyphenolic compounds, inhibit adhesion molecule expression and the adhesion of monocytes to endothelial cells, and also suppress cell inflammation, transformation, proliferation, survival, invasion, and angiogenesis [38-40]. Free radicals induce cellular damage and are involved in several human diseases such as cancer, atherosclerosis, and inflammatory disorders, and polyphenols tend to reduce mutagenic activity and oxygen-free radicals [41]. Since the initiation and progress of carcinogenesis involves mutations of DNA, the chemical alteration of DNA bases is believed to be a crucial factor. As a consequence of increased oxidative stress, DNA oxidation damage can occur with ROS, leading to mispairing of DNA bases or DNA strand breaks. ROS are generated endogenously from cellular metabolism and inflammatory responses or by exposure to exogenous agents such as ionizing radiation and xenobiotics [42].

In our study, the inhibition percentages of tested plants ranged from 74.51% (BA) to 91.45% (MA) with acidic methanolic extract concentrations at 25 μg/mL (Figure 1A). MA plants had a value of 985.73 (91.95% inhibition percentage) for tail moment at 25 μg/mL of acidic methanolic extracts (Figure 1B). The results in inhibition percentage of tail DNA% were not similar to the results in inhibition percentage of tail moment among treated samples. The MA plant extract was most effective against DNA single strand breaks in tail DNA%, while HC plant extract was most effective against DNA single strand breaks in tail moment (Figure 1A and 1B). In addition, HC plant water extracts exhibited 11.14% tail DNA% (Figure 2A) and 1255.40 (92.19% inhibition percentage) tail moment at the 25 μg/mL dose (Figure 2B). The inhibition percentage of tail DNA% results was similar to the results of the inhibition percentage of tail moment among treated samples. HC plant extracts not only had the highest Trolox equivalent (Table 4), but were also the most effective against DNA single strand breaks induced by H_2_O_2_ in human lymphocytes (Figure 1), indicating that it contains polyphenol (19.82 mg gallic acid/g DW), myricetin (146.67 μg/g DW), quercetin (3200.00 μg/g DW), and kaempferol (53.33 μg/g DW) (Tables 1 and 2). To some extent, the observed efficacy of the extracts against DNA damage can be attributed to specific flavonol constituents. The high levels of quercetin and morin are believed to account for the high DNA protective potential of HC and MA since quercetin has also been identified as an efficient reducer of DNA damage in Caco-2 cells [43]. Morin from *Psidium guajava* was effective in increasing cell viability, decreasing ROS levels, and preventing DNA fragmentation upon exposure to high glucose levels in primary rat hepatocyte cultures [44]. The antioxidant activity of polyphenolic compounds in different species showed higher polyphenolic content and antioxidant activity in all species, demonstrating that the tested species are a potent source of novel bioactive compounds with a wide range of medicinal properties. In particular, they have significant free radical scavenging activity. Our present study demonstrates that, among the six investigated species, the higher content of polyphenols, flavonols, and antioxidant properties in HC and MA plants may be the reason for their wide medicinal use. Both species can be used as potent medicinal herbs for novel bioactive compounds with high free radical scavenging activity, and extracts of these plants may been attractive alternative for managing oxidative stress-induced liver injury and drug-induced gastric ulcer [45,46]. Recently, Gargouri *et al*. [47] demonstrated that quercetin could protect against dimethoate-induced oxidative stress by decreasing lipid peroxidation and protein oxidation, and increasing superoxide dismutase and catalase activities in human lymphocytes. The herbaceous plant extracts in our study may increase antioxidant enzyme activities to protect against H_2_O_2_-induced DNA damage in human lymphocytes.

## Conclusion

Polyphenol-rich extracts from the tested plants effectively diminish DNA oxidation damage. This preventive effectiveness is attributable to the induction of cellular defenses rather than the radical scavenging activity of polyphenol and flavonols, and might well contribute to the reported health benefits of herbals. The contents of these bioactive compounds in MA and HC extracts can explain their antioxidant activity, and there exists a relationship between the content of polyphenol and flavonol to antioxidant activity. This is the first report suggestion that MA and HC plants have abundant antioxidants with strong antioxidant activity, and consequently can protect DNA in lymphocytes from oxidative damage.

## Abbreviations

ABTS: 2,2-azino-bis-(3-ethylbenzothiazoline-6-sulfonicacid); DPPH: 1, 1-diphenyl-2-picrylhydrazine; HPLC: High-performance liquid chromatography; TEAC: Trolox equivalent antioxidant capacity; TR: Trolox.

## Competing interests

The authors declare that they have no competing interests.

## Authors’ contributions

KHL prepared the extracts and carried out all the experimental process. PYC designed the current project, supervised the work and wrote the manuscript. YYY worked closely with KCL and MYH in the laboratory to carry out the experiments. HFL and HSL evaluated the data and edited the manuscript. CMY participated in statistical analysis. All the authors read and approved the final manuscript.
